# *Strobilanthes crispus* Extract Reduces Respiratory Exchange Ratio in Obese Mice Fed High Fat and Low Fat Diets

**DOI:** 10.21315/mjms2018.25.6.5

**Published:** 2018-12-28

**Authors:** Norhasnida Zawawi, Maznah Ismail

**Affiliations:** 1 Department of Food Science, Faculty of Food Science and Technology, Universiti Putra Malaysia, Malaysia; 2 Laboratory of Molecular Biomedicine, Institute of Bioscience, Universiti Putra Malaysia, Malaysia

**Keywords:** Strobilanthes crispus, LDLr knockout mice, obesity, respiratory exchange ratio, high-fat diet

## Abstract

**Background:**

*Strobilanthes crispus* (*S. crispus*) leaves were traditionally consumed for its body weight lowering effect. In this study, we investigated the anti-obesity effect of *S. crispus* leaves extract (SCE).

**Methods:**

Mice (*n* = 48) were fed high-fat diet (HFD) for 25 weeks to induce obesity, after which half were maintained on HFD and half switched to low-fat diet (LFD)while they were given normal water (H_2_O) or 0.1% (w/v) SCE in water at week 0–4 which was increased to 1% (w/v) at week 5–9. Effects of treatment with SCE were compared between HFDH_2_O, HFDSCE, LFDH_2_O and LFDSCE groups. Respiratory exchange ratios (RER) were measured at weeks 0, 5 and 10. Food, water intake and body weight were measured weekly. Plasma lipid profile and organ weights were determined at week 10.

**Results:**

SCE had significantly reduced RER at week 9 (*P* = 0.011). Food intake, body weight, and abdominal adipose tissue weight were not altered by SCE at weeks 5 and 10. However, significant increase in plasma and liver cholesterol (*P* < 0.050) was observed.

**Conclusion:**

Our findings suggest that SCE induced lipolysis and body fat oxidation and increased energy expenditure. Further studies in other animal models should be done to confirm the consistency of these results.

## Introduction

Obesity has reached epidemic proportions across the world. In 2016, WHO estimates that about 1.9 billion adults aged 18 years were overweight and at least 650 million adults were obese worldwide. The worldwide prevalence of obesity nearly tripled between 1975 and 2016. The prevalence of obesity continues to rise in many parts of the world (1). This is worrying because obesity increases the prevalence of many health hazards such as coronary artery disease, type 2 diabetes (2), hypertension (3), dyslipidaemia (4), osteoarthritis (5), obstructive sleep apnea (6), and depression (7).

The search for obesity treatments became popular since the mid-19th century when industrialisation has made obesity a prevalent problem (8). Vinegar and cabbage soup became one of the earliest widely touted obesity cures with the common rationale that the acidic makeup of these food literally chew up fat, but their success as weight management agents has more to do with psychological phenomenon than any unique chemical properties of the individual foods (8). Today, health functions of well-known micronutrients as well as traditional ethnic plant foods and herbal extracts are studied towards the development of functional, health promoting foods which includes the treatment of obesity. Although not designated as vitamins, there is a large group of compounds in fruits, vegetables, teas, and herbal extract which might not be essential throughout life or cause clinically manifested deficiencies but are essential for health and well-being of adults and the elderly population (9). For the treatment of obesity, natural products have been known to act as lipase inhibitors, metabolic stimulants, appetite suppressants, starch blockers/nutrient absorption inhibitors, glucose/insulin metabolism regulators, lipid metabolic regulators, adipogenesis inhibitors, apoptosis inducers, lipolysis inducers, and energy expenditure stimulators (10–11).

The *Strobilanthes crispus* ZII 109 (L.) Bremek or *Saricocalyx crispus* ZII 109 (L.) Bremek (Acanthaceae) plant is native to areas from Madagascar to Indonesia and is commonly known as ‘daun picah beling’ in Jakarta or ‘enyoh kelo’, ‘kecibeling’, or ‘kejibeling’ in Java (12). It was first recorded by Thomas Anderson (1832–1970) who classified the plant under Spermatophyta (flowering plants and Gymnosperma). *Strobilanthes* (cone head) was named from the combination of strobilos which means flower (13) and crispus which is phyllostachyus or spike-like leaf (phyllo means leaf, and stachyus means spike) (14). The conjunction of the names leads to the meaningful definition of the physical plant. Although there is very little record of this plant being used for medicinal purposes, a study in Indonesia found that an infusion of the dried leaves of *Strobilanthes crispus* (*S*. *crispus*) has been used as anti-diabetic, diuretic, antilytic, and laxative (12).

Numerous naturally-occurring compounds have been proposed as treatment for weight loss via enhanced energy expenditure including caffeine (15), capsaicin (16), and catechins such as epigallocatechin and epigallcatechin gallate (EGCG) (17). Natural products have also been found to have the potential for mobilising lipids by stimulating lipolysis in adipocytes which can lead to weight loss for people with obesity (18). Lipolysis in adipocytes was found to be increased by docosahexaenoic acid (DHA) in fish oil (19) and raspberry ketone from red raspberry (20).

This study investigated the effect of *Strobilanthes crispus* extract (SCE) on energy expenditure and lipolysis in the high-fat diet induced obese LDLr knockout mice.

## Materials and Methods

### Preparation of S. crispus Extract (SCE)

*S. crispus* leaves (oven dried at 40 °C overnight) were bought from the Cedar Biotea Sdn. Bhd., Pulau Pinang, Malaysia. Leaves were grounded to powder using a coffee grinder (CG100, KENWOOD, UK). Powdered leaves were stored in glass bottles and kept at −20 °C before use. For extraction, 100 g of powdered leaves was soaked and stirred continuously with chloroform-methanol (5:3, 500 mL of chloroform and 300 mL of methanol) for 12 h. This mixture was then filtered with Whatman filter paper no.4, and solvent mixture containing the extract was collected into a round bottomed flask. The flask was then connected to a rotary evaporator (Rotavapor R-3000, Buchi, Switzerland) with water bath set at 37 °C to remove majority of the solvents, until the volume reached approximately 50 mL. To evaporate the remaining solvents, crude extract was then divided into two pre-weighed conical tubes and dried under a continuous flow of nitrogen gas. To prepare the extract in water, distilled water was added to the extract paste, and the mixture was then sonicated.

### Mice

Obesity was induced in 39 LDLr KO mice by giving high-fat diet (HFD-60% kcal fat, 5.24 kcal/g, Research Diets D12492) for 24 weeks (week 26 to week 2). In addition, 10 LDLr KO mice were given low-fat diet (LFD-normal chow, 17% kcal fat, 3.3 kcal/g Harlan Teklad 2018) as control. These diets were provided by RenaSci (Biocity, Nottingham, UK). In total, 49 male LDL-receptor knockout (LDLr KO) mice aged 35 weeks, weighing 45 g–60 g at baseline were received. Mice were housed in an air-conditioned room (temperature was set to a constant 27 °C) with a 12 h-day-night cycle (lights off at 19:00, on at 07:00). HFD, LFD and SCE in water were available ad libitum to the mice, and bottles were replaced every 2 days. Bodyweight of mice, food, and water intake were measured weekly. The duration of all treatments was 10 weeks. All animal procedures were approved by the University of Nottingham Local Ethical Review Committee and were carried out in accordance with the UK Animals (Scientific Procedures) Act 1986.

### Diets

Mice were fed either HFD (Research Diets D12492 provided by Research Diets, Inc., New Brunswick, NJ, USA) or LFD (2018 Teklad Global 18% Protein Rodent Diet provided by Harlan Laboratories, Inc., Indianapolis, IN, USA). The energy provided by the macronutrients of the HFD was 20% from protein, 60% from fat, and 20% from carbohydrates, for a total of 5.24 kcal g^−1^. The kilocalories provided by the macronutrients of the LFD were 23% from protein, 17% from fat, and 60% from carbohydrates, for a total of 3.3 kcal g^−1^.

### Experimental Design

**Figure 1:**
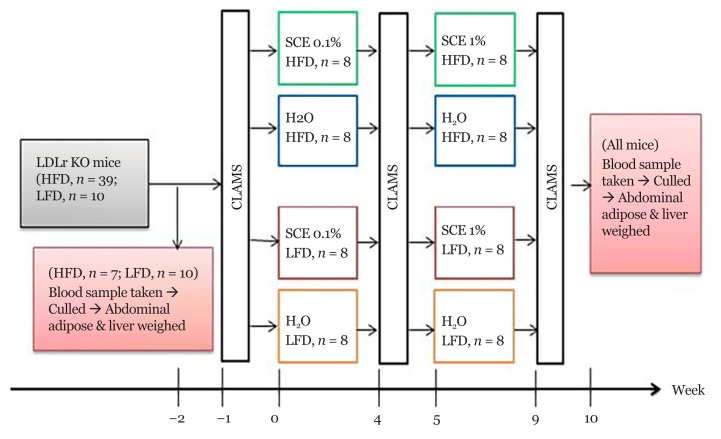
Experimental design

The experimental design is presented in Figure 1. At week 2 (baseline), 7 mice given the HFD and 10 mice given LFD were culled to obtain basal organ weights, plasma lipid levels, and liver lipid levels. The remaining mice were individually housed and acclimatised for one week.

At week 0, animals were randomly allocated into one of four treatment groups: i) the H_2_O HFD group (*n* = 8) were maintained on the HFD and given normal drinking water; ii) the SCE HFD group (*n* = 8) were maintained on HFD and given 0.1% *S. crispus* extract (SCE) in their drinking water for weeks 0–5 followed by 1% SCE for weeks 5–10; iii) the H_2_O LFD group were switched to LFD and given normal drinking water; and iv) the SCE LFD group (*n* = 8) were switched to LFD and given 0.1% SCE in their drinking water for weeks 0–5, followed by 1% SCE for weeks 5–10.

Sample size was determined using the Resource Equation Method (21). According to this method, a value “E” is measured which is nothing but the degree of freedom of analysis of variance (ANOVA). The value of E should lie between 10 and 20. If “E” is less than 10 then adding more animals will increase the chance of getting more significant result, but if it is more than 20, then adding more animals will not increase the chance of getting significant results. Although this method is based on ANOVA, it is applicable to all animal experiments. Any sample size which keeps “E” between 10 and 20 should be considered as adequate. “E” was measured by following formula:

E=total number of animals-total number of groups

### Oxygen Consumption, Carbon Dioxide and Respiratory Exchange Ratio

An Oxymax Comprehensive Lab Animal Monitoring System (CLAMS) was used on weeks 1, 4 and 9 of the study to measure 24 h profiles of oxygen consumption (VO_2_), carbon dioxide production (VCO_2_), respiratory exchange ratio (RER), motor activity, and heat production (HP). In the CLAMS (Columbus Instruments, Ohio, USA), mice were placed individually in metabolic cages equipped with oxygen sensors to measure VO_2,_ carbon dioxide sensors to measure VCO_2_ and infrared beams to determine motor activity. RER was automatically calculated by the software and expressed as VCO_2_/VO_2_. After the mice were acclimatised to the new environment for a day, data were taken for 24 h. The values for each of these parameters were then averaged for each individual mouse.

### Plasma Measurements

Blood was obtained from mice at week 10 by cardiac puncture after the administration of pentobarbitone. All mice were fasted overnight before blood sample collections. Whole blood samples were collected in EDTA-containing tubes and centrifuged at 12500 rpm for 1 h at 4 °C. Plasma was then collected and stored at −80 °C. Plasma triglyceride and glycerol were measured using a Serum Triglyceride Determination Kit (TR0100, Sigma, Missouri USA). Plasma cholesterol was measured using Infinity Total Cholesterol reagent (TR13421, Thermo Scientific, UK). Plasma glucose was measured using Infinity Glucose Oxidase reagent (TR15221, Thermo Scientific, UK).

### Total Carcass Lipid Extraction

To measure total lipid content, individual mice carcasses were weighed in aluminium containers (with lid) and freeze dried. Two grams of each sample was weighed and the total lipid content was determined with an automated Rapid Soxhlet Extraction system run according to the official method of AOAC for determination of crude fat (991.36).

### Statistical Analysis

The statistical software Genstat 10.0 was used and values were expressed as means (SEM). For biochemical profiles, body, and organ weights, two-way ANOVA was used. Comparisons for two groups were done using Independent *T*-test. All other analysis was done using repeated measure analysis of variance with no blocking (a model formula where the underlying structure of the design was not defined). Residual plots were used for checking the normality and the equal variance assumptions of the ANOVA. Normal distribution was assumed. Significance was taken as *P* < 0.05.

## Results

### Intake of Food, Water and Energy, and Energy Efficiency

**Figure 2:**
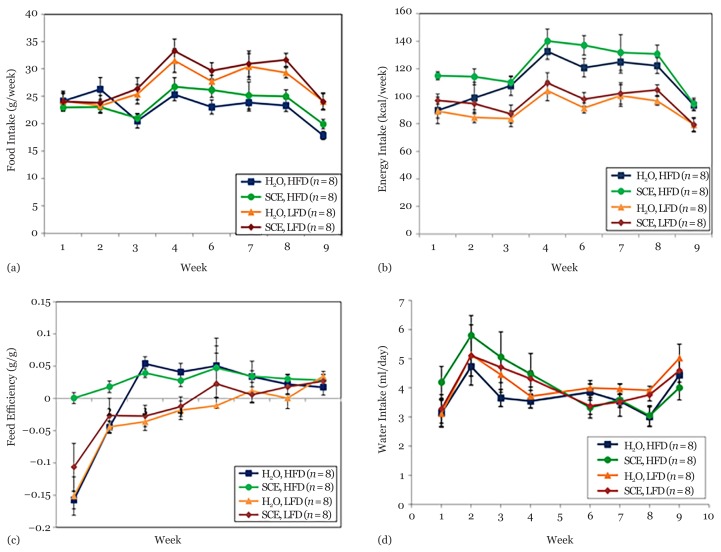
Food intake, energy intake, feed efficiency and water intake

Figure 2 shows the mean food intake (g/week) consumed in the home cages. Data at weeks 5 and 10 were not included because after animals were housed in the CLAMS at weeks 4 and 9, their food intake was reduced the following week. There were significant effects of week (*P* < 0.001) and diet (*P* < 0.001) but no interaction (*P* = 0.118). Intakes of LFD were higher than the intakes of HFD with mean food intake in HFD group across all weeks being 23.28 g, whereas the mean intake in LFD group across all weeks was 27.8 g. SCE treatment had no effect on food intake (*P* = 0.130).

### Energy Intake

Figure 2 shows the mean energy intake (kcal/week) from diet consumed in the home cages. Data at weeks 5 and 10 were not included because after animals were housed in the CLAMS at weeks 4 and 9, their food intake was reduced the following week. There was a significant interaction between week and diet (*P* = 0.012) where energy intake was found to gradually increase from week 4 to week 8, particularly in the HFD groups before coming down at week 9. LFD clearly reduces the energy intake (*P* < 0.001). Mean energy intake in the HFD group was 17.19 kcal/week, whereas the mean energy intake in the LFD group across all weeks was 12.88 kcal/week. SCE treatment tended to increase energy intake slightly (*P* = 0.082).

### Feed Efficiency

Figure 2 shows the mean feed efficiency [bodyweight gain (g)/food intake (g)] in all groups. Data at weeks 5 and 10 were not included because after animals were housed in the CLAMS at weeks 4 and 9, their body weight and food intake was reduced the following week. There was a significant interaction between week and diet (*P* < 0.001). LFD clearly reduced the feed efficiency (*P* < 0.001) particularly between weeks 1 and 6. Mean feed efficiency across all weeks in HFD group was 0.04 g/g, whereas the average of LFD group across all weeks was −0.03 g/g. SCE treatment did not have any effect on feed efficiency (*P* = 0.701).

### Water Intake

Figure 2 shows the mean SCE or water intake (mL/week) consumed in the home cages of each group. Data at weeks 5 and 10 were not included because after animals were housed in the CLAMS at weeks 4 and 9, their water intake was inaccurate in the following week. No significant effect of diet (*P* = 0.495) was found on water intake. However, there was a significant week × extract interaction (*P* = 0.025) on water intake indicating that mean water intake was higher in the SCE groups at the start of the study but lower at the end. This may cause the treated animals to receive less of the *n* 1% (w/w) SCE after the dose was increased at week 5.

### Body Weight Changes

**Figure 3:**
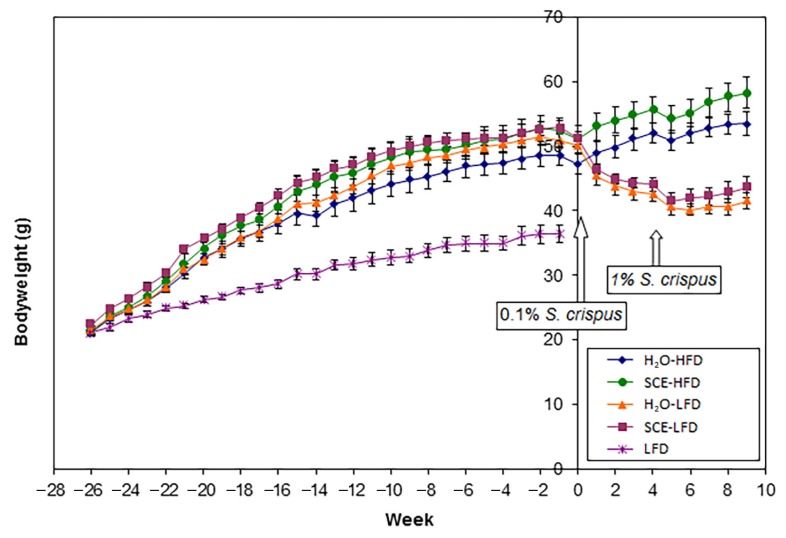
Bodyweights before and after treatment with *S*. *crispus* extract (SCE)

Figure 3 shows the changes in bodyweight from the start of treatment with HFD and after treatment with SCE (0.1% and 1%) with or without the LFD. The bodyweight of the HFD groups significantly increased with time (*P* < 0.001) compared with the LFD group such that at week 1, the HFD fed groups were 1.5 fold heavier than the LFD fed group. There were no significant differences (*P* = 0.327) in bodyweights at week 1 between the groups given HFD and then divided into H_2_O, HFD, SCE, HFD, H_2_O LFD and SCE, LFD groups.

At week 0, half the mice were switched to LFD and this significantly decreased bodyweight, whereas the mice maintained on HFD continued to increase in bodyweight (*P* < 0.001 for time × diet interaction). Treatment with SCE tended to increase bodyweight, but this was not statistically significant (*P* = 0.072). Data for weeks 5 and 10 were not included in the statistical analysis, as bodyweights of all animals were found to have reduced after the 48 h in the CLAMS.

### RER in the CLAMS

**Figure 4:**
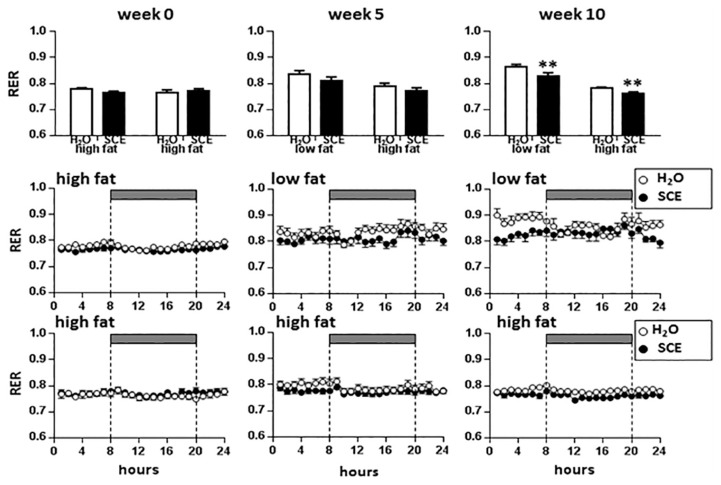
Respiratory Exchange Ratio (RER) during CLAMS at weeks 0, 5 and 10

Figure 4 shows the changes in RER at weeks 0, 5 and 10 observed over 24h. At week 0, as expected, there were no effects of diet (*P* = 0.592) or extract (*P* = 0.693) on RER, but there was a significant effect of time of day (*P* = 0.001). The range for RER values at week 0 was 0.73 to 0.83 for all groups. At week 5, there was a significant diet × time of day interaction (*P* = 0.013) with LFD increasing the RER and reducing the circadian rhythm, but no effect of extract on RER was observed at this stage (*P* = 0.111). At week 10, there was no longer effect of time of day on RER (*P* = 0.225), but there were significant effects of diet (*P* < 0.001) and extract (*P* = 0.011) with RER being increased by LFD, but reduced by SCE.

### Plasma Glucose and Lipid Concentrations

Plasma glucose and lipid concentrations were analysed from plasma obtained at baseline (week 2) and at the end of the study (week 10). For data at baseline (Table 1), samples were taken from control obese mice (*n* = 7) given HF diet and control lean mice (*n* = 10) given LFD for 25 weeks. For data at week 10 of treatment (Table 2), samples were taken from the diet and SCE treated groups.

At baseline (Table 1), HFD was found to have significantly increased plasma glucose, total cholesterol, and glycerol concentrations (all *P* < 0.001) compared with LFD. Plasma triglyceride concentrations were numerically higher in HFD, but this was not statistically significant (*P* = 0.376). At week 10 of treatment (Table 2), LFD was found to significantly reduce plasma glucose, total cholesterol, glycerol, and triglyceride concentrations (all *P* < 0.001). There was a significant interaction between diet and extract (*P* = 0.013) on plasma total cholesterol concentrations due to SCE increasing the total cholesterol concentrations but only in HFD group. SCE also significantly increased (*P* = 0.032) plasma glycerol concentrations in both HFD and LFD groups.

### Liver Lipid Content

Liver lipid contents were analysed from livers obtained at baseline at the end of the study (week 10 of treatment) which were homogenised in isopropanol. For data at baseline (Table 1), samples were taken from the livers of control obese mice (*n* = 7) given HFD and control lean mice (*n* = 10) given LFD for 25 weeks. For data at week 10 of treatment (Table 2), samples were taken from diet and SCE treated groups.

At baseline (Table 1), significant increases in liver triglycerides and cholesterol content (all *P* < 0.001) were found in the HFD group and this was maintained at week 10 of treatment (Table 2), all *P* < 0.001). SCE increased liver cholesterol contents (Table 2) but only in the HFD group (*P* = 0.032 for diet and extract interaction).

### Carcass Lipid, Bodyweight and Organ Weight

At baseline and at the end of the study (week 10 of treatments), mice were weighed, culled and then their abdominal fat, liver and heart were dissected out and weighed, and the remaining carcasses were homogenised and analysed for total lipid content. For data at baseline (Table 1), samples were taken from control obese mice (*n* = 7) given HFD and control lean mice (*n* = 10) given LFD for 25 weeks. For data at week 10 of treatment (Table 2), samples were taken from the diet and SCE treated groups.

At baseline (Table 1), HFD was found to significantly increase body weight (*P* < 0.001), abdominal fat weight (*P* = 0.002), carcass lipid content (*P* < 0.001), liver weight (*P* < 0.001), and heart weight corrected for bodyweight (*P* = 0.022). Similarly, at week 10 (Table 2), HFD significantly increased body weight (*P* < 0.001), abdominal fat weight (*P* < 0.001), % abdominal fat (*P* < 0.001), carcass lipid content (*P* < 0.001), liver weight (*P* < 0.001), % liver weight (*P* = 0.001) and % heart weight (*P* = 0.022) compared with LFD. The only significant effects of SCE were the increase in liver weight (*P* = 0.019) and % liver weight (*P* = 0.029), the decrease in % heart weight (*P* = 0.047), and the tendency to increase body weight (*P* = 0.072) and abdominal fat weight (*P* = 0.079).

## Discussion

### HFD-Induced Obesity in LDLr KO Mice

Traditionally, leptin-deficient (ob/ob) and leptin receptor-deficient (db/db) mice are used for studies on obesity but their utility in studying atherosclerosis is limited (22). On the other hand, the LDLr KO mouse has been used extensively as a research model to investigate pathways involved with lipid metabolism and as an in vivo model for atherosclerosis (23–25). On high fat or high cholesterol ‘Western type’ diets containing 21% fat and 0.15% added cholesterol, LDLr KO mice develop severe hyperlipidemia and extensive atherosclerosis (26). Furthermore, LDLr KO mice have also been reported to exhibit diet-induced weight gain with high fat diet feeding (27) making it viable to use as a model for obesity research (28–30). Thus, in this study, the LDLr KO mouse model was used to study the effect of SCE (0.1 and 1%w/w) on diet-induced obesity in the presence of hyperlipidemia.

HFD feeding allows the characterisation of obesity development and the evaluation of anti-obesity interventions in an in vivo experimental setting that is pathophysiologically similar to the human disease (31). Many attributes of obesity were found in the HFD-fed group at baseline before any treatments were started.

The HFD was confirmed to induce obesity. As a rule, experimental animals eating LFD do not become obese (32). Development of obesity in animals eating HFD is the expected outcome (33). It has also been found by Cha and colleagues (34) that when animals are exposed to several concentrations of dietary fat, there is a dose-response curve with a threshold at about 25% dietary fat. This suggests that the concentration of dietary fat needs to exceed 25% in the diet before obesity develops. In this experiment, the 34.9 (% per g) or 60% kcal of fat in the HFD successfully induced obesity.

Mice have been the predominant model of energy homeostasis and obesity in humans (35) because the attributes of diet-induced obese mice as a result of excessive energy intake and weight gain induced by the use of energy-dense foods are similar to human subjects. However, it must be noted that the environment of the mice studied was fully controlled (they were individually housed in cages that limit physical activity (in rooms controlled for temperature and humidity with food available nearby). This might be the reason for increased abdominal and carcass fat and liver weights of HFD-fed groups and the higher level of plasma glucose, total cholesterol, and glycerol concentrations in the LFD-fed controls (Table 1). Liver triglycerides and cholesterol had also risen above the concentrations in the LFD-fed group (Table 1). Human obesity is a more complex multifactorial condition that has been related to excessive food consumption, low energy expenditure, genetics, gender, age, socio-economic status, ethnicity, educational concentration, smoking status and many other factors (35). Thus, while controlled conditions in the diet-induced obese mice were necessary to enable experimental control, they do not necessarily mimic the normal human condition.

### Change to a LFD Reduces Obesity in LDLr KO Mice

In the home cages, the bodyweights of LF groups (LFD, H_2_O and LFD, SCE) immediately decreased after 1 week consumption of LFD and continued to decrease until week 5 when their bodyweights became stabilised (Figure 3). The loss of bodyweight was a result of reduced total body fat, liver, and heart weights (Table 2). Even though food intake was found to be significantly increased by the LFD, the total energy intake of LFD-fed groups was significantly lower than HFD-fed groups and this resulted in decreased feed efficiency which contributed to the decrease in bodyweights. LFD was also found to improve (reduce) plasma glucose and lipid concentrations and also liver lipid contents (Table 2). Plasma glucose, total cholesterol, trigylcerides, and glycerol concentrations all decreased in LFD-fed groups. Similarly, liver triglyceride and cholesterol contents were also reduced by LFD. Only water intake was not altered by LFD.

In the CLAMS, LFD was found to have no significant effect on oxygen consumption, but carbon dioxide release was found to have increased at some timepoints at week 4 and significantly increased at all timepoints at week 9. Since RER is the ratio of VCO_2_ (total carbon dioxide production) to VO_2_ (total oxygen consumption), obviously the increased VCO_2_ at weeks 4 and 9 lead to the significant increase of RER by LFD. The RER value is used to indicate nutrient utilisation (36). When the RER value is shifted closer to 1, this indicates reliance on carbohydrate as the major energy substrate. On the other hand, if the RER value is shifted closer to 0.7, this indicates a major reliance on fat oxidation (37). In our study, when HFD was switched to LFD which had 40% higher calories from carbohydrate, the RER increased indicating that the animals increased their utilisation of carbohydrates. LFD was found to have no effect on the frequency of meals taken by the animals at week 9 even though their food intake increased in the home cages. Unfortunately, a technical problem meant that the data at week 4 were lost.

It is well known that when obese subjects (rodents and humans) are given an LFD ad libitum, weight loss is induced and plasma lipid profile (38–40) is improved, and our findings confirm this. However, weight reduction does not always reverse obesity condition (32). Previous research confirmed that the number of fat cells of both mice and rats increase after eating HFD for an extended time, and these cells remain after dietary fat is reduced (41–43).

### S. crispus Extract Decreased RER without Altering Food Intake or Other Variables in LDLr KO Mice

From the results shown in Table 2, SCE may induce adipose tissue lipolysis (increased plasma glycerol), though not at a high enough concentration to significantly reduce adipose tissue weights or total body fat content at the time measured. However, the increase in plasma and liver cholesterol could contribute to the development of arteriosclerosis.

In the CLAMS, SCE was found to significantly decrease the RER value at weeks 5 and 10. The decreased RER suggests that SCE increased lipid oxidation which may be a positive effect. Previous research (44) showed that obese Sprague-Dawley rats fed a normal chow diet and treated with 1% *S. crispus* extract (in drinking water–approximately 1 g/kg BW/day) have reduced body weight, adipose tissue and liver weights and tended to lower body weight gain, plasma leptin and fasting glucose levels compared to control obese rats fed the same normal chow diet without showing toxic effects. The lipolysis rate of plasma glycerol (per abdominal fat in gram) in the treated groups was also found to be higher. This suggests that the extract acted as a lipolysis inducer in the treated rats. However, it is not known whether the lipolysis effect was through energy expenditure as this was not measured.

Most weight loss agents fall broadly into two biological effect areas: those that affect energy intake (appetite suppressants and malabsorption agents) and those that affect energy expenditure (45). In this case, however, the energy expenditure increased due to the decrease of RER value.

However, another comparable study which used Molokheiya leaves (*Corchorus olitorius* L.) to reduce diet-induced obesity in HFD fed LDLr KO mice had more convincing results, with body weight gains, epididymal adipose tissue, and liver weights reduced, as well as plasma glucose and triglyceride concentrations lowered and activation of beta oxidation detected. However, the experiment is not exactly similar as the mice in this study were not obese at the start of the experiment and treatments were only done for 8 weeks (46).

## Conclusion

Obesity was successfully induced in LDLr KO mice with many attributes of obesity comparable with that of human obesity. LFD successfully improved the obesity condition by lowering energy intake without increasing energy expenditure. SCE was found to increase lipolysis and fat oxidation in obese LDLr KO mice but not enough to be suggested as an anti-obesity agent. However, further studies could be done to investigate the effects in other in vivo models to confirm the consistency of these results.

## Figures and Tables

**Table 1:** Biochemical profiles and organ weights at week 0

Diet	High Fat (HFD) (*n* = 7)	High Fat (HFD) (*n* = 7)	*P*-value
Body weight, BW (g)	48.84 (3.06)	36.48 (4.18)	**< 0.001**
Abdominal fat weight (g)	3.43 (0.36)	2.29 (0.76)	**0.002**
% Abdominal fat (g/100g BW)	7.03 (0.56)	6.15 (1.61)	0.191
Carcass lipid (% dry weight)	71.50 (2.15)	54.00 (5.69)	**< 0.001**
Liver weight (g)	2.33 (0.21)	1.70 (0.26)	**< 0.001**
% Liver weight (g/100g BW)	4.77 (0.39	4.64 (0.34)	0.478
Heart weight (g)	0.20 (0.04)	0.19 (0.03)	0.308
% Heart weight (g/100g)	0.41 (0.06)	0.51 (0.09)	**0.022**

Glucose (mg/ml)	3.39 (0.18)	2.39 (0.18)	**< 0.001**
Total Cholesterol (mM)	25.10 (8.25)	10.80 (2.70)	**< 0.001**
Triglycerides (μg/ml)	2637.00 (0.10)	2178.00 (0.13)	0.376
Glycerol (μg/ml)	1510.00 (0.63)	375.00 (0.12)	**< 0.001**

Triglycerides (μmol/g)	245.10 (42.10)	44.00 (12.39)	**< 0.001**
Cholesterol (μmol/g)	15.19 (0.22)	10.16 (0.88)	**< 0.001**

**Table 2:** Biochemical profiles and organ weights at week 10

Diet (D)	High-fat (HFD)		Low-fat (HFD)			*P*-value	
			
Extract (E)	H_2_O (*n* = 8)	SCE (*n* = 7)	H_2_O (*n* = 8)	SCE (*n* = 8)	Diet (D)	Extract (E)	D × E
Body weight, BW (g)	52.8 (6.55)	58.4 (5.86)	40.03 (3.11)	42.53 (5.52)	**< 0.001**	0.072	0.413
Abdominal fat weight (g)	3.51 (0.62)	3.99 (0.42)	2.01 (0.42)	2.22 (0.65)	**< 0.001**	0.079	0.493
% Abdominal fat weight (g/100g BW)	6.65 (1.04)	6.86 (0.72)	4.99 (0.74)	5.12 (1.14)	**< 0.001**	0.607	0.909
Carcass lipid (% dry weight)	71.64 (9.00)	78.16 (11.28)	59.62 (9.58)	61.77 (5.98)	**< 0.001**	0.117	0.407
Liver weight (g)	2.96 (0.80)	3.81 (0.69)	2.03 (0.36)	2.23 (0.48)	**< 0.001**	**0.019**	0.134
% Liver weight (g/100g BW)	5.52 (0.90)	6.50 (0.69)	5.03 (0.53)	5.19 (0.60)	**0.001**	**0.029**	0.108
Heart weight (g)	0.20 (0.02)	0.19 (0.07)	0.19 (0.02)	0.18 (0.03)	0.327	0.536	0.967
% Heart weight (g/100g)	0.38 (0.04)	0.32 (0.10)	0.46 (0.04)	0.42 (0.08)	**0.001**	**0.047**	0.761
Plasma Glucose (mg/ml)	3.28 (0.43)	3.50 (0.38)	2.26 (0.17)	2.52 (0.24)	**< 0.001**	0.249	0.923
Plasma Cholesterol (mM)	27.24 (6.96)	36.90 (3.54)	11.07 (1.81)	12.56 (3.16)	< 0.001	0.001	**0.013**
Plasma Triglycerides (μg/ml)	2463.00 (0.10)	2817.00 (0.05)	1224. 00 (0.05)	1516.00 (0.06)	**< 0.001**	0.141	0.884
Plasma Glycerol (μg/ml)	1602.00 (0.93)	23.24 (0.83)	189 (0.09)	428.00 (0.42)	**< 0.001**	**0.032**	0.266
Liver Triglycerides (μmol/g)	317.00 (79.03)	416.00 (46.35)	76.00 (30.98)	174.00 (71.76	**< 0.001**	**0.001**	0.979
Liver Cholesterol (μmol/g)	17.64 (0.45)	23.81 (0.22)	11.68 (0.14)	10.58 (0.49)	< 0.001	0.127	**0.032**

Table shows the mean values with standard deviation (in brackets) of plasma lipid and liver contents, carcass lipid, liver and heart weights at Week 10 in 4 groups of LDLr-KO mice given either high-fat diet combined with either *S. crispus* extract (SCE) of not (Control). Two-way ANOVA was used to compare between groups. *P*-values are expressed for Diet (D), Extract (E) and interaction between D and E.
